# Modulation of Aβ42 in vivo by γ-secretase modulator in primates and humans

**DOI:** 10.1186/s13195-015-0137-y

**Published:** 2015-08-05

**Authors:** I-Fang Ling, Todd E. Golde, Douglas R. Galasko, Edward H. Koo

**Affiliations:** Department of Neurosciences, University of California, La Jolla, San Diego, CA USA; Department of Neuroscience, University of Florida, College of Medicine, Gainesville, FL USA; Departments of Medicine and Physiology, Yong Loo Lin School of Medicine, National University of Singapore, Singapore, Singapore

## Abstract

**Introduction:**

Ibuprofen is one of the nonsteroidal anti-inflammatory drugs that have been shown to selectively lower pathogenic amyloid beta-peptide (Aβ)42 without impairing overall γ-secretase activity in vitro. This γ-secretase modulator (GSM) activity has been hypothesized to contribute to the reduction in risk of developing Alzheimer’s disease in chronic users of nonsteroidal anti-inflammatory drugs. However, it is unclear whether ibuprofen, within therapeutic dosing range, demonstrates GSM activity in humans. In this study, we evaluated the effects of ibuprofen and a second-generation GSM, GSM-1, on Aβ levels in cerebrospinal fluid and plasma of young nonhuman primates and humans.

**Methods:**

Five to seven conscious cynomolgus monkeys (*Macaca fascicularis*) were nontreated or treated with 30 mg/kg GSM-1 or 50 or 100 mg/kg ibuprofen and the plasma and cerebrospinal fluid were sampled at −8, 0 (baseline or right before treatment), 2, 4, 6, 8, 12, and 24 h postdosing. In addition, sixteen healthy human subjects were randomly assigned to receive either placebo or 800 mg ibuprofen given by intravenous administration and plasma were collected at 0 (before drug infusion), 0.5, 1, 2, 4, 6, 8, 10, and 24 h after dosing.

**Results:**

A single dose of GSM-1 (30 mg/kg) decreased the ratio of Aβ42 to Aβ40 to 60 % in plasma and the ratio of Aβ42 to total Aβ to 65 % in cerebrospinal fluid from baseline to postdosing in monkeys. However, no significant changes were detected following ibuprofen treatment at 100 mg/kg. Consistent with the results from nonhuman primates, ibuprofen did not alter plasma Aβ levels in human volunteers after a single 800 mg dose.

**Conclusions:**

GSM-1 exerted potent lowering of the ratio of Aβ42 to Aβ40 in nonhuman primates but the hypothesized GSM activity of ibuprofen could not be demonstrated in nonhuman primates and humans after acute dosing.

## Introduction

Alzheimer’s disease (AD) is the most common age-associated neurodegenerative disease and is accompanied by hippocampal and brain atrophy and is manifested clinically with memory and cognitive impairments [1]. Pathologically, AD is characterized by deposition of extracellular amyloid beta-peptide (Aβ) in senile plaques, intracellular neurofibrillary tangles, and loss of synapses and neurons [2]. The cause of AD is still unclear. Much research focuses on the amyloid cascade hypothesis, which states that aggregated forms of Aβ initiate a cascade of events that result in the full neurodegenerative features of AD [3, 4]. Aβ is derived by sequential proteolysis of the amyloid precursor protein (APP) by β- and γ-secretases to generate peptides of 36–42 amino acids in length [5, 6]. The longer but minor species, Aβ42, is widely believed to represent a more pathogenic species as it aggregates faster than the shorter Aβ40 species and demonstrates toxicity in vitro and in vivo [7, 8]. Mutations in *PSEN1*, *PSEN2* and *APP* genes, causing early onset familial AD (FAD) have been shown to alter Aβ production, with the majority of *PSEN* mutations elevating the ratio of Aβ42/Aβ40 [9–11]. At present there is no effective treatment for AD and current medications are mildly effective in treating the symptoms but do not show disease-modifying effects [12].

Because γ-secretase cleavage is the final step in Aβ production, inhibition or modulation of γ-secretase serves a logical therapeutic target for AD. In contrast to the nondiscriminant inhibition of γ-secretase inhibitors (GSI), γ-secretase modulators (GSMs) are thought to be intrinsically safer as they target the pathogenic Aβ42 species without inhibiting normal APP processing or cleavage of other γ-secretase substrates, such as Notch and ErbB4 [13, 14]. GSMs increase the processivity of γ-secretase to promote further cleavage of Aβ42 peptides, resulting in decreased Aβ42 and increased shorter species, such as Aβ37 and Aβ38, thereby reducing the ratio of Aβ42/Aβ40 [15]. GSM activity was first observed in a subset of nonsteroidal anti-inflammatory drugs (NSAIDs), such as ibuprofen, indomethacin and sulindac sulfide, when used at high doses, an observation that might explain the epidemiological link between NSAID use and AD risk reduction [16–18]. Since the initial description of the GSM activity of certain NSAIDs, numerous GSMs with more potent activity have been reported [19–21]. However, it is unclear whether NSAIDs exhibit GSM activity in humans as this has never been tested. Thus, if such compounds were to be used prophylactically as potential preventive treatment, it is important to establish whether the NSAIDs with GSM activity in vitro behave similarly in vivo*.*

Although GSMs have shown efficacy in preclinical models, tarenflurbil, the *R*-enantiomer of flurbiprofen was shown not to be effective in a late-phase human clinical trial possibly due to lack of potency or brain penetration [22, 23]. Because newer GSMs have not yet demonstrated clinical efficacy, we chose to examine whether ibuprofen has GSM activity in nonhuman primates and humans following acute dosing. Ibuprofen is one of the NSAIDs with GSM activity in preclinical studies and chronic use has been linked to a reduction in AD risk [16, 18, 24–27]. However, it is unclear whether the drug, within the therapeutic dosing range, can modulate Aβ42 levels in vivo in humans. Consequently, in this study, we evaluated the effects of ibuprofen and a potent second-generation GSM, GSM-1 [28], on plasma and cerebrospinal fluid (CSF) Aβ levels in young cynomolgus monkeys, a model that has been used to examine GSI efficacy in vivo [29]. In addition, we took advantage of the recent US Food and Drug Administration (FDA) approval of an intravenous (IV) formulation of ibuprofen (Caldolor) to examine the effect of a single high dose of ibuprofen on plasma Aβ levels in humans. In contrast to GSM-1, we failed to detect any GSM activity of ibuprofen on plasma and CSF Aβ42 levels in nonhuman primates or plasma Aβ42 levels in humans.

## Methods

### Reagents and antibodies

All compounds and reagents were from commercial venders as follows: IV-ibuprofen (Caldolor; Cumberland Pharmaceuticals, Nashville, TN, USA); ibuprofen (Nurofen; Ibuprofen B.P. 200 mg; Reckitt Benckiser, Slough, UK); Aβ38 and Aβ40 enzyme-linked immunosorbent assay (ELISA) kits (IBL-International, Toronto, Canada); INNOTEST Aβ42 kit (Innogenetics, Alpharetta, GA, USA); MSD Aβ Triplex kit (Meso Scale Discovery, Gaithersburg, MD, USA); and Ibuprofen ELISA kit (Neogen, Lexington, KY, USA). GSM-1 was custom synthesized at Mayo Clinic (Jacksonville, FL, USA) by following published procedures [30] and verified by a combination of thin-layer chromatography (TLC), nuclear magnetic resonance (NMR) and electrospray ionization (ESI) analyses. The in vitro efficacy has been confirmed in previous publications [31, 32]. Antibodies included Ab9 (Mayo Clinic), 82E1 (IBL-International), 4G8-biotin (Covance, Princeton, NJ, USA), streptavidin-HRP (Jackson ImmunoResearch, West Grove, PA, USA), Aβ42 specific antibodies MM26-2.1.3 (Mayo Clinic), and 6E10-HRP (Covance).

### Cell culture

Chinese hamster ovary (CHO) cells stably expressing wild-type APP751 (APP-WT) and CHO cells stably expressing APP751 with the Val717Phe familial AD mutation (APP-V717F) were maintained in Dulbecco’s modified Eagle’s medium (DMEM) supplemented with 10 % fetal bovine serum, 50 U/ml penicillin and 50 μg/ml streptomycin at 37 °C in a humidified 5 % CO_2_/95 % air atmosphere.

### Drug treatments in cynomolgus monkeys

All procedures related to the use of animals were approved by the Institutional Animal Care and Use Committee (IACUC) of Maccine Pte Ltd (Singapore). A total of seven, non-naive female cynomolgus macaques (*Macaca fascicularis*), aged 2.5–5 years, were studied. A cisterna magna catheter and port system was surgically implanted in each monkey [33] and animals were allowed recover for 2–3 weeks prior to treatment. Following recovery, the animals were studied sequentially over the subsequent weeks under different conditions, which were changed on a weekly basis. In the first week, no treatment was given and the CSF and plasma were sampled from the monkeys at −8, 0 (baseline), 2, 4, 6, 8, 12, and 24 h. In the following weeks, the animals were treated with GSM-1 (30 mg/kg), IV-ibuprofen (50 mg/kg), or ibuprofen (100 mg/kg) with a 7-day rest/washout period between each treatment condition.

IV-ibuprofen was diluted with saline to 4 mg/ml and IV infused to achieve the dose of 50 mg/kg. Ibuprofen was supplied as a 200 mg caplet. Each animal was provided with one caplet of 200 mg ibuprofen and one capsule containing the weighed amount of the grounded ibuprofen caplet to meet the required dose of 100 mg/kg. Ibuprofen was administered by using an appropriate catheter tube. GSM-1 was prepared by dissolving in 5 % (v/v) ethanol, 10 % (v/v) solutol HS-15 and 85 % (v/v) Milli-Q water on the day of dosing. Oral administration of GSM-1 was achieved by gavage.

### Human study

The study was approved by Human Research Protections Program and the Institutional Review Board at the University of California, San Diego, and carried out at the Shiley-Marcos Alzheimer’s Disease Research Center and Clinical Translational Research Institute at the University of California, San Diego. Subjects were recruited and screened. Exclusion criteria included contraindication to blood draw, chronic major psychiatric disorders, history of peptic ulcer or upper gastrointestinal bleed, significant medical illness, hypersensitivity/allergy to ibuprofen or other NSAID drugs, and regular use of aspirin or NSAIDs. All subjects provided written informed consent to participate in this study.

Sixteen eligible healthy subjects, 6 men and 10 women from 21 to 35 years of age, participated in this study. After an overnight fast, the subjects were randomized to receive 800 mg IV-ibuprofen or placebo. Plasma was sampled at time 0 (before drug infusion), 0.5, 1, 2, 4, 6, 8, 10, and 24 h after dosing.

### Sandwich ELISA

Quantification of total Aβ and Aβ42 peptides was carried out with sensitive ELISAs. For total Aβ, Ab9 antibody (20 μg/ml), which recognizes Aβ1–16, was used as capture and 4G8-biotin (20 μg/ml) and strepavidin-HRP were used as reporter. Aβ42 was measured by the Aβ42 end-specific MM26-2.1.3 antibody (50 μg/ml) [34] as capture antibody and 6E10-HRP (1 μg/ml) as reporter. 4G8 and 6E10 recognize Aβ17–24 and Aβ1–16, respectively. CSF Aβ38 was measured by a commercial Aβ38 ELISA kit (IBL), following manufacturer’s instructions. Plasma Aβ species were measured by either Aβ40 (IBL), Aβ42 (Innotest) or Aβ Triplex (MSD) kit, following manufacturer’s instructions. Plasma levels of ibuprofen were measured by a commercial ELISA system (Neogen), following manufacturer’s instructions.

### Statistical analysis

The effects of drugs on CSF and plasma Aβ ratios in monkeys and plasma Aβ ratios in humans were analyzed by a two-way analysis of variance with a post hoc Bonferroni posttest (GraphPad Prism, La Jolla, CA, USA).

## Results

### GSM activity of ibuprofen and GSM-1 in vitro

**Fig. 1:**
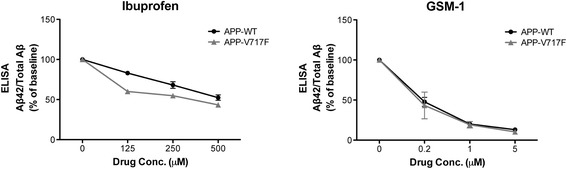
The efficacy of ibuprofen and GSM-1 on selectively decreasing Aβ42 in vitro. Total amyloid beta-peptide (Aβ) and Aβ42 levels in conditioned media from cells stably expressing wild-type APP751 (APP-WT) and cells stably expressing APP751 with the Val717Phe familial AD mutation (APP-V717F) after drug incubation for 24 h were evaluated by enzyme-linked immunosorbent assays (ELISAs) and ratios were compared. All experiments were repeated at least twice. Results are presented as mean ± range. *GSM* gamma-secretase modulator

This study was designed to test the physiological activity of two GSMs in primates following acute dosing: a NSAID ibuprofen and GSM-1, an experimental compound. Before evaluating the effects in vivo, we first confirmed their GSM activity in vitro. CHO cells stably expressing APP-WT or APP-V717F were incubated with ibuprofen or GSM-1 for 24 h. Conditioned media were collected and Aβ42 and total Aβ levels were quantified by ELISA analyses. Both ibuprofen and GSM-1 significantly reduced the ratio of Aβ42 to total Aβ in a dose-dependent manner, indicating the modulation of γ-secretase activity (*p* < 0.001; Fig. 1). As expected, GSM-1 showed greater potency with 50 % reduction at 0.2 μM whereas ibuprofen required 250–500 μM to reach similar level of Aβ42 changes (Fig. 1).

### Effects of ibuprofen and GSM-1 on Aβ in cynomolgus monkeys

**Fig. 2:**
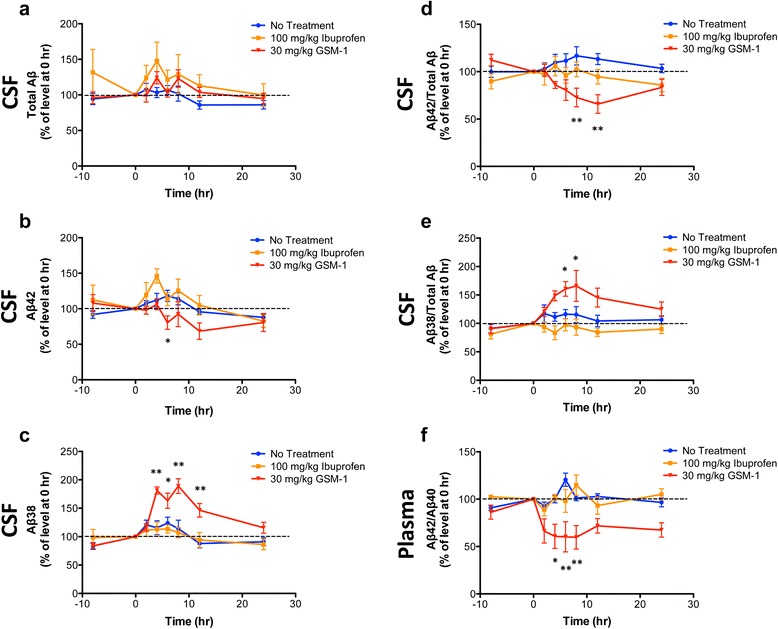
The effects of ibuprofen and GSM-1 on CSF and plasma Aβ levels in cynomolgus monkeys. Cerebrospinal fluid (CSF) and plasma samples were collected at indicated time points and amyloid beta-peptide (Aβ) levels were measured. Levels of total Aβ (**a**), Aβ42 (**b**) and Aβ38 (**c**) were compared in CSF samples. Ratios of Aβ42 to total Aβ (**d**) and Aβ38 to total Aβ (**e**) were compared in CSF samples and ratios of Aβ42/Aβ40 were compared in plasma samples (**f**). The levels and ratios were normalized to baseline (time 0 or before drug dosing) and shown as percentages of baseline. Seven monkeys were used in the no-treatment group. However, the catheters in two animals did not maintain patency during the study period. Therefore, fewer animals received the single dose of GSM-1 (n = 6) or ibuprofen (n = 5). Results are presented as mean ± SEM; *p < 0.05, **p < 0.001. *GSM* gamma-secretase modulator

Seven young cynomolgus monkeys were first studied without drug treatment to determine the basal Aβ levels over a 32-hour period. The CSF and plasma samples were collected at −8, 0 (baseline), 2, 4, 6, 8, 12 and 24 h after baseline. Aβ42, Aβ38 and total Aβ levels in CSF samples were measured. During the observation period, there was variation in Aβ levels in untreated animals (Fig. 2a–c) without a clear diurnal pattern, consistent with the reports from catheter studies in humans [35]. Animals were then treated with drugs in subsequent weeks sequentially. Between each treatment, all animals were rested for 1 week to allow drug washout. In drug-treated animals, GSM-1 significantly reduced Aβ42 and increased Aβ38 levels (both *p* < 0.001), but did not change total Aβ levels (Fig. 2a–c). Maximal reduction of ~32 % in Aβ42 levels (from 267.1 ± 60.0 (mean ± SD) to 183.5 ± 96.1 pg/mL) was noted at 12 h postdosing (Fig. 2b). Meanwhile, Aβ38 levels increased ~90 % maximally (from 1033.3 ± 300.0 to 1954.8 ± 698.6 pg/mL; Fig. 2c) at 8 h postdosing. GSM-1 decreased the ratio of Aβ42 to total Aβ up to 35 % at 12 h after administration (*p* < 0.001; Fig. 2d) and increased the ratio of Aβ38 to total Aβ maximally by 70 % after 8 h (*p* < 0.05; Fig. 2e). In light of the CSF results, the effects of GSM-1 were mirrored in plasma. Specifically, the ratio of Aβ42/Aβ40 in plasma was markedly decreased (*p* < 0.001; Fig. 2f). This was evident as early as 2 h following drug administration and plateaued starting at 4 h. GSM-1 reduced the ratio of Aβ42/Aβ40 up to 40 % at 6 h after dosing. These changes in plasma Aβ were more rapid than were observed in CSF, where the maximum reduction was delayed to 8–12 h, but the magnitude of reduction was similar (Fig. 2d,f).

In contrast to GSM-1, no changes could be detected in any of the Aβ levels or ratios in either CSF or plasma following ibuprofen administration at either 50 mg/kg (data not shown) or 100 mg/kg (Fig. 2). Specifically, there was no reduction in the ratio of Aβ42 to total Aβ nor an increase in the ratio of Aβ38 to total Aβ in CSF and no change in the ratio of Aβ42/Aβ40 in plasma after ibuprofen treatment (Fig. 2d–f). In sum, these results showed that a single-dose administration of GSM-1 potently modulated Aβ production. In contrast, a single high dose of ibuprofen did not result in any detectable changes in Aβ production in monkeys that would be consistent with γ-secretase modulation.

### Effects of ibuprofen on Aβ in humans

**Fig. 3:**
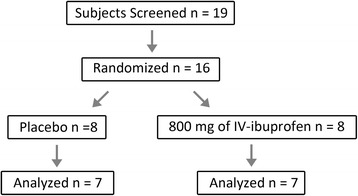
Participation flow chart for the human study. Nineteen subjects were recruited and screened. Three subjects were excluded from the study: two due to high blood creatinine level and invisible veins, respectively, and one declined to participate after screening. Samples from two subjects were excluded for analysis due to technical reasons. *IV* intravenous

**Fig. 4:**
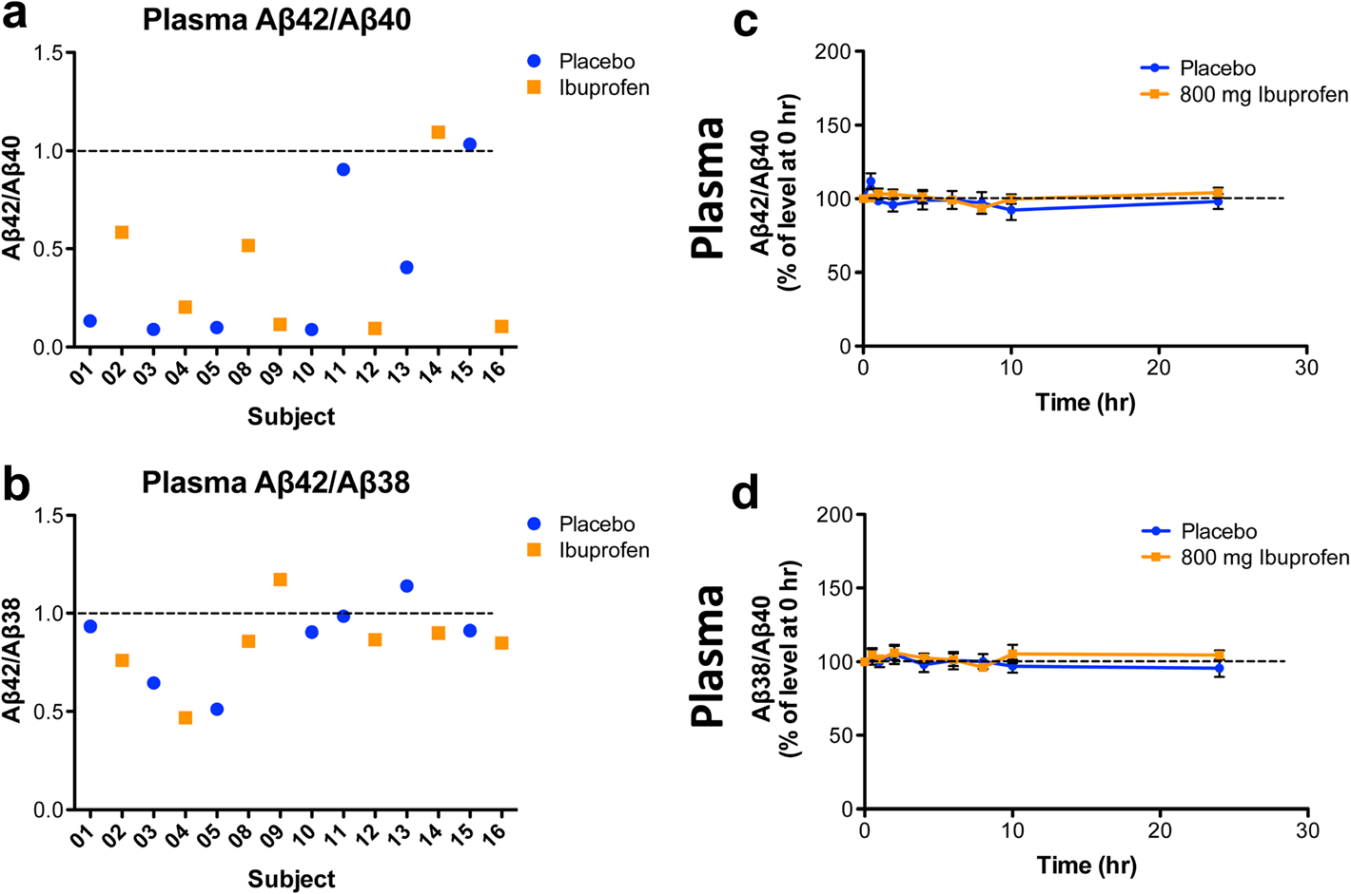
The effect of a single dose of 800 mg IV-ibuprofen on Aβ levels in humans. Plasma samples were collected at indicated time points and amyloid beta-peptide (Aβ) levels were analyzed. Ratios of Aβ42/Aβ40 (**a**) and Aβ42/Aβ38 (**b**) at baseline (before infusion) were compared among subjects. Then ratios of Aβ42/Aβ40 (**c**) and Aβ38/Aβ40 (**d**) between placebo and ibuprofen groups were compared. Results are presented as mean ± SEM

We next evaluated the effects of IV-ibuprofen or placebo on plasma Aβ levels in humans. Nineteen subjects were screened and sixteen eligible healthy subjects were enrolled in the study (Fig. 3). With eight subjects in each group, we anticipated being able to detect changes of 20 % or more in plasma Aβ42 levels if ibuprofen showed GSM activity based on a GSI study [36]. Subjects were randomly assigned to receive either placebo or 800 mg IV-ibuprofen. The latter dose reflects the highest FDA-approved single human dose given four times daily (total of 3.2 g per day). Given the plasma results of GSM-1 treatment in monkeys, blood samples were drawn at baseline (before drug infusion), 0.5, 1, 2, 4, 6, 8, 10, and 24 h after dosing. During the study period, no adverse events were observed in subjects. The levels of Aβ38, Aβ40 and Aβ42 in plasma samples were measured. Subject demographics and baseline Aβ levels are summarized in Table 1. Baseline plasma levels of Aβ peptides showed substantial variability between subjects. The Aβ42 levels ranged from 23.2 to 1524.5 pg/mL, and the Aβ40 levels ranged from 229.2 to 1392.2 pg/ml. When the baseline ratios of Aβ42/Aβ40 were compared, most subjects reflected the generally minor contribution of Aβ42 to Aβ40 with ratios of approximately 0.1, but several subjects (#11, 14 and 15) showed relatively higher Aβ42 levels with ratios of approximately 1 (Fig. 4a). In contrast, the ratios of Aβ42/Aβ38 ranged from 0.5 to 1 with the majority around 1 (Fig. 4b). Comparing either the ratios of Aβ42/Aβ40 and Aβ38/Aβ40 in plasma in subjects who received ibuprofen versus placebo, no changes could be detected after drug treatment (Fig. 4c,d). These results demonstrated that a single dose of 800 mg ibuprofen did not result in any acute changes in Aβ production in humans, a finding consistent with the observations from nonhuman primates.

**Table 1:** Demographic data and subject characteristics at baseline (n = 7 for each group)

	Placebo	Ibuprofen
Age (mean ± SD)	27.4 ± 5.5	25.7 ± 3.6
Sex	3 females, 4 males	5 females, 2 males
Aβ38 Level (pg/mL)	199.1 ± 276.0	344.6 ± 603.5
(mean ± SD (lowest; highest))	(25.7; 746.8)	(25.6; 1694.4)
Aβ40 Level (pg/mL)	348.2 ± 152.8	468.0 ± 409.5
(mean ± SD (lowest; highest))	(229.2; 659.1)	(259.9; 1392.2)
Aβ42 Level (pg/mL)	183.8 ± 257.6	295.5 ± 547.3
(mean ± SD (lowest; highest))	(23.2; 681.4)	(27.5; 1524.5)

**Fig. 5:**
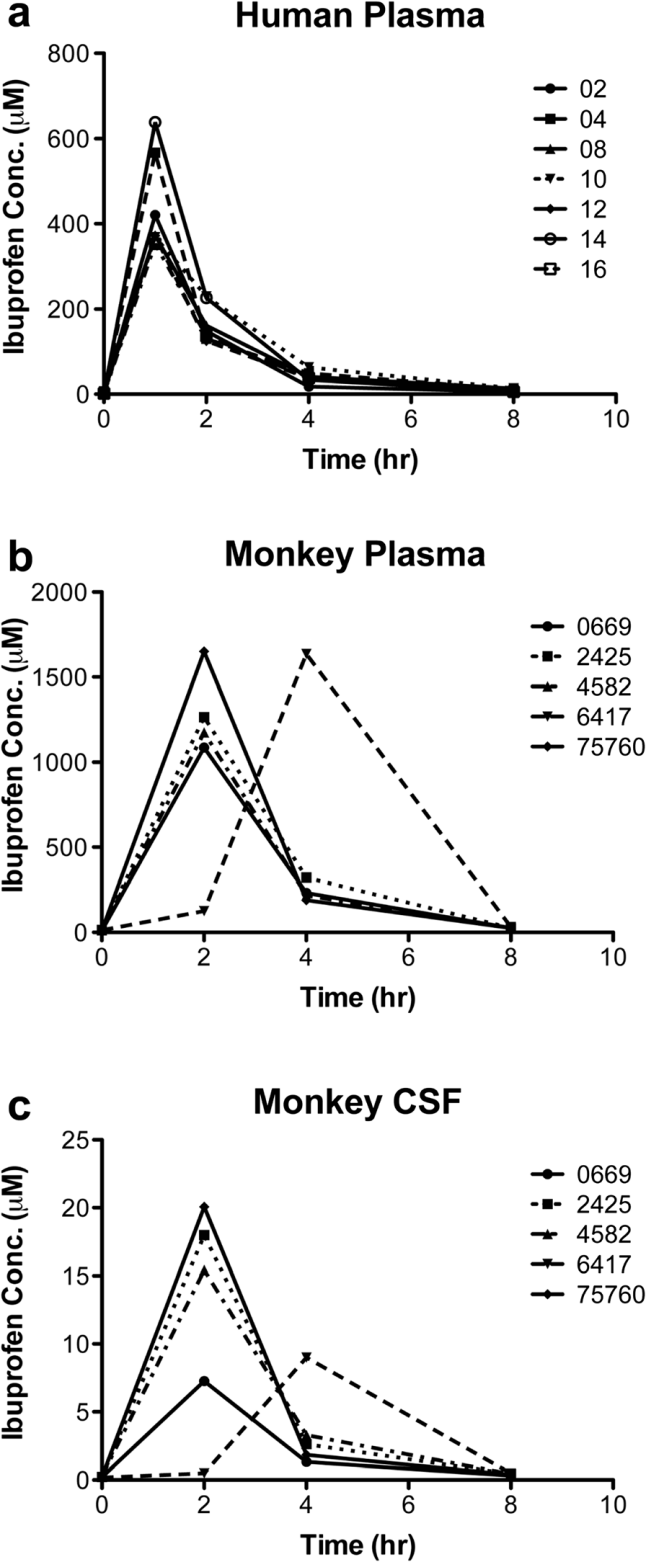
Ibuprofen concentrations in human and monkey samples after dosing. Ibuprofen concentrations were evaluated by ELISAs. **a** Plasma samples from seven human subjects who received 800 mg of IV-ibuprofen were analyzed. **b** Plasma and **c** cerebrospinal fluid (CSF) samples from five monkeys treated with 100 mg/kg ibuprofen were evaluated

The negative results in plasma after ibuprofen treatment in both humans and monkeys were unexpected. Although IV administration should have resulted in near complete systemic drug delivery, it is important to confirm adequate drug levels in plasma in humans. Because the half-life of ibuprofen is approximately 2 h in plasma [37], drug levels were measured in samples at 0, 1, 2, 4, and 8 h after infusion. For comparison, we also analyzed the monkey CSF and plasma samples at 0, 2, 4, and 8 hours after oral dosing with 100 mg/kg ibuprofen. In humans, the highest plasma levels of ibuprofen were seen at 1 hour, the first time sampled, and returned to baseline levels by 8 h (Fig. 5a). The maximum average concentration of total ibuprofen (bound and unbound) (C_max total_) was 441.63 ± 113.84 (mean ± SD) μM (Table 2). Because ibuprofen has been reported to be ~99 % bound to plasma proteins [38], free unbound concentration of ibuprofen was estimated to be approximately 4.4 μM. In monkeys, ibuprofen concentrations were highest at 2 h, again the first sampled time point, after oral administration in all but one animal in both plasma and CSF (Fig. 5b,c). The C_max total_ in CSF was 12.24 μM, which is ~ 1 % of the C_max total_ in plasma (1060.33 μM) (Table 2). Comparing the area under curve from 0 to 8 h, the calculated CSF:plasma ratio was about 0.01 in monkeys after a single dose of 100 mg/kg ibuprofen (Table 2), consistent with the generally low brain penetration of NSAIDs [39, 40]. Finally, interleukin (IL)-1β and IL-6 levels were assayed from human plasma and, not surprisingly, there were no differences between control and ibuprofen-treated groups (data not shown) because there is no documented suppression of these inflammatory markers by ibuprofen in healthy individuals [41].

**Table 2:** Pharmacokinetics of ibuprofen in humans (800 mg) and monkeys (100 mg/kg) after acute treatment

	Human (n = 7)	Monkey (n = 5)	
	Plasma	CSF	Plasma
C_max total_ (μM)^a^	441.63 ± 113.84	12.24 ± 8.18	1060.33 ± 565.25
AUC_0–8h_ (μM*h)	831.44 ± 155.92	36.36 ± 11.75	3744.8 ± 924.16
CSF:plasma ratio		0.01 ± 0.004	

^a^Total concentration (bound and unbound). *AUC*_*0–8h*_ area under the curve from 0 to 8 h, *C*_*max*_ maximum average concentration, *CSF* cerebrospinal fluid

## Discussion

In this study, we sought to test whether ibuprofen demonstrates any GSM activity in plasma and CSF in nonhuman primates and humans following acute dosing. Contrary to previous in vitro studies, a single high dose of ibuprofen did not modulate Aβ levels as would be predicted if the compound has γ-secretase modulating activity in vivo at the dose given acutely. At a high dose, but within the therapeutic dosing range (i.e., 800 mg IV-ibuprofen), there were no changes in Aβ levels in human plasma. Even higher doses in nonhuman primates, 100 mg/kg, failed to demonstrate Aβ42 reduction in CSF or plasma. In contrast, a second-generation GSM, GSM-1, demonstrated potent changes in Aβ42 and Aβ38 levels in CSF and plasma in monkeys indicative of γ-secretase modulation. These latter findings showed that γ-secretase complex can be modulated in primates in a manner consistent with prototypic GSM activity first defined in vitro and in rodents.

Epidemiological studies have consistently shown that chronic usage of NSAIDs reduced the risk of AD and, further, that the protective effects are linked with the duration of NSAID use [17, 18, 42]. A recent study using a Veterans Administration database reported that ibuprofen, one of the three most commonly used NSAIDs together with naproxen and indomethacin, had the strongest protective effect against AD when used for more than 5 years [18]. However, there was no correlation between NSAIDs that had GSM activity versus those that did not [43]. In a 1-year treatment trial of mild to moderate AD individuals, there was no benefit of 400 mg ibuprofen given twice daily, but a sub-analysis of apoE4 individuals suggested slowing in cognitive decline [44]. Other treatment trials with NSAIDs have either been negative or demonstrated equivocal benefit [45–48].

Unresolved in all the observational studies are the mechanisms by which NSAID use may lower the risk for developing AD, if this happens [49]. A number of mechanisms have been proposed but none have been rigorously tested [50]. In this study, we tested whether ibuprofen may act as a GSM and reduce production of the pathogenic Aβ42 peptides through this mechanism. In preclinical studies, subacute treatment with 50 mg/kg ibuprofen for 3 days significantly reduced Aβ42 but not Aβ40 levels in the Tg2576 line of APP transgenic mice [16, 51]. Chronic treatment with ibuprofen decreased amyloid load in the brain accompanied by reduction in all Aβ species, and rescued memory deficits in the same APP transgenic mouse line [16, 24, 26, 27], but the mechanisms for these effects were not established. Furthermore, we are not aware of a study in rodents that examined the changes in Aβ peptides after acute treatment with ibuprofen to demonstrate GSM activity, nor has the GSM activity of ibuprofen been examined directly in primates. Results from this study showed that, contrary to expectations, a single high dose of ibuprofen did not reduce Aβ42 levels in monkeys or humans (Figs. 2 and 4). The maximum average concentrations of total ibuprofen detected from plasma in monkeys and human subjects were 1060.33 μM and 441.63 μM, respectively (Table 2). Although the assay used in this study may be less quantitative than other methods, such as liquid chromatography-tandem mass spectrometry, the drug levels were within the range of published reports [37]. Further, we did not measure free versus bound concentration of the drug. However, because ibuprofen is about 99 % bound to plasma proteins in humans [38], the estimated unbound concentration of ibuprofen was ~4.4 μM. In mice treated with 50 mg/kg ibuprofen daily for 3 days and reported to demonstrate GSM activity, the average plasma drug concentration was 43 μM, 2 h after the last dose [51]. Because the unbound fraction of ibuprofen in rodents has been reported to be ~5.5 % [52], the estimated unbound ibuprofen concentration in that study would be ~2.37 μM, a level that is comparable to what we detected in humans. Consequently, the lack of effect of ibuprofen in humans and monkeys cannot be easily attributable to insufficient drug exposure, at least if treatment for 3 days in mice is comparable to the single acute treatment given in the present study. Another potential explanation for the negative results is that, because of lower efficacy, ibuprofen may require more sustained treatment, as was done in the APP transgenic mouse line. However, in view of the robust effects of GSM-1 in monkeys, this reasoning is perhaps less convincing. Therefore, our results suggested that if subchronic to chronic treatment of ibuprofen were effective in decreasing brain Aβ levels in primates, it may not be due to direct γ-secretase modulation, but possibly acting in combination with other mechanisms, such as lowering pro-inflammatory cytokines to decrease APP expression and Aβ accumulation, increasing anti-inflammatory cytokines to facilitate Aβ clearance, or anti-aggregation properties to reduce Aβ fibril formation [50]. Interestingly, in the 5XFAD mice with aggressive early amyloid pathology, 3 months of treatment with ibuprofen resulted in an increase rather than decrease in soluble Aβ levels and worsening of behavioral performance even in the presence of demonstrated reduction in inflammatory responses. This study in rodents called into question the beneficial effects of anti-inflammatory treatments targeting the cyclooxygenase pathway [53]. Taken together, the effects of ibuprofen are complicated. If indeed they are beneficial in preventing AD in humans, the mechanism of risk reduction may occur through multiple pathways that have thus far not been adequately tested in preclinical or clinical settings.

In stark contrast to ibuprofen, the second-generation GSM-1 significantly decreased Aβ42 ratios in CSF and plasma in monkeys. GSM-1 is a NSAID-derived carboxylic acid-containing GSM originally developed by Merck by modulating compound structure to reduce lipophilicity [19–21, 30]. The drug efficacy in transgenic mice was first reported by Page and colleagues where a single dose of GSM-1 given at even 3 mg/kg lowered brain Aβ42 by 25 % and, at 30 mg/kg, brain Aβ42 was reduced by 70 % in APP transgenic mice. As would be expected, Aβ38 levels were concomitantly increased [28]. Consistent with these rodent studies, we observed a 40 % reduction in ratio of Aβ42/Aβ40 in plasma 4 h after dosing, whereas CSF ratio of Aβ42 to total Aβ was decreased by 35 % 12 h after treatment in monkeys (Fig. 2d,f). At the same time, changes in Aβ38 levels appeared more rapidly and were more sustained (Fig. 2e). While the reciprocal Aβ42 and Aβ38 changes are one of the “signatures” of NSAID-based GSMs, it is unclear whether these alterations are mechanistically linked or occur independently. The uncoupling of the two events had been reported in the setting of presenilin 1 (PS1) or PS2 mutations [28, 54]. In addition, a recent study reported that a novel GSM decreased both Aβ42 and Aβ38 levels while increasing Aβ37 and Aβ39 peptides [55]. These results suggested this coupling is not necessarily obligatory.

As expected, the second generation GSM-1 is much more potent than ibuprofen in vitro with an ED_50_ of GSM-1 of approximately 200 nM, compared to 250–500 μM for ibuprofen (Fig. 1). Although not reported for GSM-1, a GSM with similar structure and comparable in vitro and in vivo GSM activity, GSM-10 h, demonstrated excellent brain penetration in an APP transgenic mouse line (TASTPM) [56]. In contrast, as estimated from the CSF to plasma ratio, ibuprofen brain penetration was about 1 % in monkeys (Table 2), a value consistent with other NSAIDs [39, 40, 51]. Furthermore, NSAIDs such as fenofibrate and flurbiprofen have been reported to bind to APP, presumably one mechanism of action underlying the NSAID-based GSMs [57]. On the other hand, GSM-1, like other second-generation compounds, binds directly to the PS1 N-terminal fragment and through this interaction is believed to alter γ-secretase processing in an allosteric manner [58–61]. Whether these differences in binding sites confer different efficacy or mode of action has not been established.

## Conclusions

This study showed that a single high dose of ibuprofen did not result in γ-secretase modulating activity in nonhuman primates and humans. It is unclear whether multiple dosing would achieve the desired Aβ42 reduction as this was not addressed in our study. However, a more potent second-generation compound, GSM-1, selectively decreased plasma and CSF Aβ42 levels in nonhuman primates in a manner consistent with the GSM class of drugs. This study also reaffirms the utility of using a nonhuman primate model to examine AD-targeted therapeutics in a more physiologically relevant system.

## Abbreviations

Aβ: Amyloid beta-peptide
AD: Alzheimer’s disease
APP: Amyloid precursor protein
APP-V717F: APP751 with Val717Phe mutation
APP-WT: Wild-type APP751
CHO: Chinese hamster ovary
C_max_: Maximum average concentration
CSF: Cerebrospinal fluid
DMEM: Dulbecco’s modified Eagle’s medium
ELISA: Enzyme-linked immunosorbent assay
ESI: Electrospray ionization
FAD: Familial Alzheimer’s disease
FDA: US Food and Drug Administration
GSI: Gamma-secretase inhibitor
GSM: Gamma-secretase modulator
IL: Interleukin
IV: Intravenous
NMR: Nuclear magnetic resonance
NSAID: Nonsteroidal anti-inflammatory drug
PS: Presenilin
TLC: Thin-layer chromatography
